# Doctors and Drug Companies: Still Cozy after All These Years

**DOI:** 10.1371/journal.pmed.1000359

**Published:** 2010-11-02

**Authors:** David Henry

**Affiliations:** 1 Institute for Clinical Evaluative Sciences, Toronto, Ontario, Canada; 2 Department of Medicine, University of Toronto, Toronto, Ontario, Canada; 3 School of Medicine and Public Health, University of Newcastle, Callaghan, New South Wales, Australia

## Abstract

David Henry discusses a research article by Geoffrey Spurling and colleagues that examined the relationship between exposure to promotional material from pharmaceutical companies and the quality, quantity, and cost of prescribing.

This Perspective discusses the following study published in *PLoS Medicine*:

Spurling GK, Mansfield PR, Montgomery BD, Lexchin J, Doust J, et al. (2010) Information from Pharmaceutical Companies and the Quality, Quantity, and Cost of Physicians' Prescribing: A Systematic Review. PLoS Med 7(10): e1000352. doi:10.1371/journal.pmed.1000352.

Geoff Spurling and colleagues report findings of a systematic review looking at the relationship between exposure to promotional material from pharmaceutical companies and the quality, quantity, and cost of prescribing. They fail to find evidence of improvements in prescribing after exposure, and find some evidence of an association with higher prescribing frequency, higher costs, or lower prescribing quality.

The relationships between doctors and drug companies are controversial and have long been scrutinized by researchers, ethicists, professional bodies, and legislators [1]. In recent years, growing concerns about these ties, and allegations of some corrupt practices, have engendered a large amount of coverage in the media and professional journals [2]–[4].

In my experience, the main concerns about close ties between companies and doctors are that 1) they lead to inappropriate prescribing that can harm patients; 2) they create divided loyalties for doctors between the health system, their patients, and manufacturing companies, which is a conflict of commitment as well as a conflict of interest; 3) they lead to use of unnecessary and expensive medications with consequent costs falling on health care systems and patients; 4) they may lead to medicalisation of human variation, i.e., “disease-mongering”; and 5) they diminish the professional standing of doctors in the eyes of the public and governments, which leads to a reduced ability to advocate for the health of patients, for the public, and on behalf of the profession.

In response to community concerns, legislators have tried to improve the transparency of the relationships between doctors and drug companies—for example, the recently passed Physicians Payments Sunshine Act in the United States, and mandatory disclosure requirements for companies in Australia [5],[6]. These require public reporting of certain types of industry-sponsored activities; in Australia this includes the nature of the sponsored meetings, the venues, any hospitality provided, and overall costs [6].

In response to widely voiced concerns, professional bodies around the world have tightened their codes of conduct, and the state of Massachusetts passed legislation banning gifts from drug and device manufacturers [7]–[9]. Drug companies are trying to reduce some of their more egregious activities, such as provision of lavish gifts and entertainment, and overly generous travel support. Recent revisions to the *Code of Practice of the Pharmaceutical Research and Manufacturers of America* specifically prohibit these activities [8]. Such activities have long been the focus of those who have questioned the relationships between doctors and drug companies. They have also been the main target of the legislative responses in the US and Australia. But, open-ended activities such as “unrestricted” research grants, “educational” grants, membership in speakers' bureaus and advisory panels, consultancies, and stock-holding could be of greater concern, through an insidious blurring of professional boundaries and obligations [10]. There is evidence that these types of ties are common among specialist physicians [11].

Underlying all of these concerns is a belief that close ties between doctors and pharmaceutical companies have been shown to create the negative effects noted at the start of this article. It is fair to ask about the evidence underpinning these beliefs. The paper by Geoffrey Spurling and colleagues in the October 2010 issue of *PLoS Medicine* addresses the question of whether drug company information has an impact on doctors' prescribing [12]. This publication is timely and important. It is a substantial update to previous work—38 of the 58 studies that were included did not feature in previous reviews. Spurling and colleagues highlight some important points. It was not possible to obtain confident summary quantitative estimates of the effects of industry activities, and they ended up expressing the overall results by doing “head counts”. The majority of studies found either an undesired effect on prescribing quality or costs, or found no effect. The lack of a quantitative summary measure is not surprising, but is regrettable, as the overview of numbers of studies rather than their results takes no account of the effect size, the sample size, or the quality of individual studies. However, the authors made an assessment of the methodological rigor of the studies included in their review. They concluded, not surprisingly, that it was low. There was a heavy reliance on cross-sectional studies and time series analyses, which are susceptible to a range of biases and order effects. There were only two randomised trials, and these were not relevant as they did not test the interventions generally used in the field by pharmaceutical manufacturers.

Spurling and colleagues made a solid assessment of the methodological quality of this literature and addressed two additional concerns—publication bias and outcome reporting bias. The former is the well-known tendency for authors to submit only positive studies for publication. Publication bias seems more common in the case of low quality non-randomised studies, the type reviewed here [13]. This is acknowledged by the authors. Tests for publication bias include funnel plot asymmetry, which requires an estimate of effect size and precision for each study, and is not possible with this literature. The authors seem to argue against their results being subject to outcome reporting bias. This has been identified as the tendency for studies to be published, but for authors to report preferentially those outcomes that changed significantly with the intervention [14]. The authors of this review found that significant associations between exposure to industry promotion and changes to measures of prescribing were more common in studies that reported a single unit of analyses than those that reported multiple units of analyses. They argue against reporting bias, but one possible explanation of their results is that the authors were selective about reporting their units of analyses, being more likely to do this when they found significant associations.

But does any of this matter? Sometimes we are forced to draw conclusions and take actions even when the supporting evidence is of a low level, as it is here. When assessing a body of evidence for harm we have to consider a number of factors, including the magnitude of the effect and the quality of the research behind the claims. But there are other dimensions, including the potential benefits of the activities and the availability of alternatives (in this case other sources of information on new pharmaceutical products). These questions, normally applied to treatments, may sit uncomfortably in a political economy where private companies have the right, indeed the obligation, to market their products effectively to health professionals.

But if industry promotional activities influence the treatments that patients receive, we should ask for evidence of benefit. If that benefit is better knowledge and more effective and safer use of medications, and commercial promotion is better at doing this than publicly funded drug information, we should be prepared to tolerate some adverse effects. If the benefits are slight, or absent, then we should have a low tolerance for any adverse effects. Spurling and colleagues may have difficulty demonstrating a strong evidentiary base for claims of harm from industry promotion, but they have done an effective job of excluding any important benefit from this relationship [13].

So why don't governments, all of whom struggle with the costs of new drugs, make greater efforts to provide unbiased prescribing information to doctors? Activity is patchy. For instance, the Australian government makes a modest but admirable attempt through funding the National Prescribing Service, and in England there is a National Prescribing Centre (NPC) with “NPC associates” in Primary Care Trusts [15],[16]. By contrast, where I live, in Ontario, Canada, neither the national nor the provincial government makes any general effort to inform doctors, or to modify prescribing practices. The pharmaceutical industry may still hold the medical profession in a warm embrace, but they don't seem to be at serious risk of being jilted in favor of other suitors.
